# Remotely sensed soil moisture to estimate savannah NDVI

**DOI:** 10.1371/journal.pone.0200328

**Published:** 2018-07-11

**Authors:** Niklas Boke-Olén, Jonas Ardö, Lars Eklundh, Thomas Holst, Veiko Lehsten

**Affiliations:** 1 Department of Physical Geography and Ecosystem Science, Lund University, Lund, Sweden; 2 Swiss Federal Institute for Forest, Snow and Landscape research (WSL), Birmensdorf, Switzerland; Pacific Northwest National Laboratory, UNITED STATES

## Abstract

Satellite derived normalized difference vegetation index (NDVI) is a common data source for monitoring regional and global ecosystem properties. In dry lands it has contributed to estimation of inter-annual and seasonal vegetation dynamics and phenology. However, due to the spectral properties of NDVI it can be affected by clouds which can introduce missing data in the time series. Remotely sensed soil moisture has in contrast to NDVI the benefit of being unaffected by clouds due to the measurements being made in the microwave domain. There is therefore a potential in combining the remotely sensed NDVI with remotely sensed soil moisture to enhance the quality and estimate the missing data. We present a step towards the usage of remotely sensed soil moisture for estimation of savannah NDVI. This was done by evaluating the European Space Agency (ESA) Climate Change Initiative (CCI) soil moisture and three of its individual products with respect to their relative performance. The individual products are from the advance scatterometer (ASCAT), Soil Moisture and Ocean Salinity (SMOS), and the Land Parameter Retrieval Model-Advanced Microwave Scanning Radiometer-Earth Observing System (LPRM-AMSR-E). Each dataset was used to simulate NDVI, which was subsequently compared to remotely sensed NDVI from MODIS. Differences in their ability to estimate NDVI indicated that, on average, CCI soil moisture differs from its individual products by showing a higher average correlation with measured NDVI. Overall NDVI modelled from CCI soil moisture gave an average correlation of 0.81 to remotely sensed NDVI which indicates its potential to be used to estimate seasonal variations in savannah NDVI. Our result shows promise for further development in using CCI soil moisture to estimate NDVI. The modelled NDVI can potentially be used together with other remotely sensed vegetation datasets to enhance the phenological information that can be acquired, thereby, improving the estimates of savannah vegetation phenology.

## Introduction

Satellite derived normalized difference vegetation index (NDVI) is useful for monitoring regional and global ecosystem properties such as ecosystem functioning [1], crop classification [2], or phenology [3]. A commonly used source of NDVI is the MODerate Resolution Imaging Spectrometer (MODIS) onboard the Terra and Aqua satellites. Each of the satellites generates daily NDVI from which a 16-day vegetation index composite product is generated. However, those MODIS-NDVI composites, and other similar products, are affected by atmospheric conditions, e.g. clouds and aerosols, which affect the quality of the composite products and generate missing data. Several methods to fill the missing data exist, using for example filters, decomposition techniques, or regression techniques [4]. There is a potential for combing remotely sensed soil moisture with other products to extract enhanced land surface information [5] or to estimate missing data. Remotely sensed soil moisture has in contrast to NDVI the benefit of being unaffected by cloud cover due to the measurements being made in the microwave domain [5, 6]. Furthermore, the temporal resolution of NDVI data has been identified as a factor restricting the accuracy in predicting the tree leaf onset for an African savannah [7]. As a first step towards using remotely sensed soil moisture to enhance NDVI data we are investigating the potential to model NDVI from remotely sensed soil moisture.

Soil moisture on its own is an important environmental variable that influences vegetation cover and energy exchange between the land surface and the atmosphere. Accurate knowledge of soil moisture at coarse scale can be useful for plant growth modelling [8], estimation of vegetation phenology, and drought monitoring [9]. Several products of remotely sensed soil moisture are currently available, partly differing in temporal coverage and post processing. One of these is the combined product from the European Space Agency (ESA), which is part of the Climate Change Initiative (CCI). The CCI soil moisture has long temporal extent (1978 to 2014) [10] and combines data from different satellite sensors to create a merged dataset [11]. In this study, we evaluate CCI soil moisture and three of its individual products with respect to their relative performance in estimating NDVI for savannahs, globally. Previous evaluation studies of remotely sensed soil moisture have focused on comparison with in-situ soil moisture at a point scale [12–16] or been carried out at local scale in small regions [17]. McNally, Shukla [9]evaluated CCI soil moisture for East Africa using modelled soil moisture and NDVI in order to assess its usefulness to be included in drought monitoring. They found that remotely sensed soil moisture can add useful information and showed on average a correlation of 0.58 between NDVI and soil moisture for the period 1992–2013. It has been found that errors in remotely sensed soil moisture from SMOS (Soil Moisture and Ocean Salinity) were affected by vegetation cover [18]. On the other hand, soil moisture estimates from AMSR-E (Advanced Microwave Scanning Radiometer-Earth Observing System) have been reported to capture the temporal variability of in-situ soil moisture well, but differed in absolute values for temperate and semi-arid regions [19]. It has also been shown that ASCAT (the advanced scatterometer), AMSR-E and SMOS in general provided good temporal agreement to the soil moisture from a land surface model for a range of Mediterranean landscapes in Northeast Spain. It has been shown that CCI soil moisture has a similar or better performance compared to its individual products, with exception to ASCAT [20]. This indicates a potential for using the combined soil moisture product, which has the benefit of a long temporal extent. To our knowledge, there exist no previous evaluation studies of CCI soil moisture with respect to estimating NDVI for savannahs.

We therefore compared the performance of CCI soil moisture with the three of its individual products, which are from the advance scatterometer (ASCAT), Soil Moisture and Ocean Salinity (SMOS), and Land Parameter Retrieval Model-Advanced Microwave Scanning Radiometer-Earth Observing System (AMSRE). The remotely sensed soil moisture was for each of the four datasets converted to NDVI using a previous developed time lagged statistical model [21] which was compared against remotely sensed NDVI from MODIS. The aim was to study the performance of the merged CCI product for estimating NDVI in comparison to using one of its individual single products. The comparison was carried out by analyzing the correlation to MODIS-NDVI for savannahs, globally.

## Methods

### NDVI estimation from soil moisture

The main soil moisture dataset included in this study was the multi-decadal merged soil moisture product being part of the Climate Change Initiative (CCI) provided by the European Space Agency. It combines data from four active and two passive sensors to create a merged dataset [11], from now on referred to as CCI (Table 1). CCI was compared to three of its individual products, one active and two passive datasets (Table 1). The active product originated from the advanced scatterometer (ASCAT) on board METOP [22, 23], from now on referred to as ASCAT. The two passive products were one from Land Parameter Retrieval Model-Advanced Microwave Scanning Radiometer-Earth Observing System (LPRM-AMSR-E) [24] and one from Soil Moisture and Ocean Salinity (SMOS) [25], from now on referred to as AMSRE and SMOS.

**Table 1:** Summary of used datasets and their temporal extent.

Dataset	Variable^a^	Temporal extent used	Resolution	Reference
AMSRE L3 D V0001	sm	2009–2010	0.25°	[24]
CCI v02.2 Combined	sm	2009–2010 & 2003–2014	0.25°	[11, 29, 30]
SMOS CLF31A	sm	2010–2011	0.15°	[25]
ASCAT WARP 5.5 Release 2.1	sm	2009–2010	0.10°	[22]
MOD13C1/MYD13C1	NDVI	2009–2011 & 2003–2014	0.05°	[28]
MCD12C1- IGBP	LC	2009	0.05°	[28]
Aridity Index	AI	1950–2000 (average)	30"	[31]
Tree Cover v1.1	TREE	2000	0.9"	[32]
Rainfall WFDEI CRU	MAP	1982–2012 (average)	0.5°	[27]

^a^sm is soil moisture; NDVI is Normalized Difference Vegetation Index; LC is land cover; TREE is tree canopy cover; MAP is mean annual precipitation

Because of differences in the temporal extents of the soil moisture datasets the longest overlapping time periods were used for the comparison; 2009–2010 for AMSRE, CCI, and ASCAT; and 2010–2011 for SMOS. The soil moisture products that contained quality information were preprocessed to remove low quality data. For CCI we removed data that were flagged as snow, temperature below zero, dense vegetation, or no model convergence. For SMOS, values flagged as snow, frost, ice, interception, rain, strong topography, open water, wetlands, urban, or coast were removed. The soil moisture datasets were temporally gap filled with a linear interpolation to create daily data. They were further smoothed with an 8-day median filter and matched to the 8-day time steps of MODIS-NDVI.

A time-lagged relationship was used to model NDVI from soil moisture (Eq 1, [21]). It uses a two time steps (16 days) time-lagged day length (dayL_2_) and soil moisture (sm_2_) combined with long term mean annual precipitation (MAP) to predict NDVI. The lagged relationship was introduced in the model to allow for a time delay in vegetation activity following a climatic event and the two-time step lag was chosen based on model performance. Furthermore, temperature was not included in the model due to its correlation with day length. Day length was calculated using pixel-center latitude and date using Herbert Glarner´s formula [26]. The parameters in Eq 1 were selected using a multiple variable regression model, for more detailed information about the model see Boke-Olén et al. [21]. The model (Eq 1) was used to estimate NDVI from each of the remotely sensed soil moisture products which were subsequently evaluated against MODIS-NDVI.

$$NDVI=0.12∙{log}_{e}(MAP∙{sm}_{2})+0.01∙{dayL}_{2}+0.22$$ (Eq 1)

MAP was derived from the Water and Global Change (WATCH) Forcing Data methodology applied to ERA-Interim data [27]. Day length was calculated from latitude and date, and all the datasets (Table 1) were averaged to a common 0.25 degree spatial resolution. Because of the extent for which the time-lagged relationship (Eq 1) was developed, we only considered savannah areas located between 60 degrees south and 40 degrees north. The land cover used to categorize savannahs was the MCD12C1-IGBP land cover classification [28].

### Evaluation

NDVI modelled with remotely sensed soil moisture products were evaluated against remotely sensed NDVI from the Moderate Resolution Imaging Spectroradiometer (MODIS, [28]). We combined the two MODIS-NDVI products MOD13C1 and MYD13C1 to produce a combined dataset with an average temporal resolution of 8-days. MODIS-NDVI data flagged as marginal, snow or ice, or cloudy was filtered out, and the dataset was spatially averaged to the common 0.25 degree resolution used throughout the study and filtered with a Savitsky-Golay filter using the same approach as Boke-Olén et al. [21] used. Since the temporal variation plays an important role when using the modelled NDVI to estimate missing data, the evaluation of the datasets was based on a correlation analysis where MODIS-NDVI was the reference.

The estimated NDVI was evaluated against MODIS-NDVI by calculating a per-pixel correlation coefficient (r) for each remotely sensed soil moisture dataset. We decided to use r instead of r^2^ to avoid the problem that negative r values (close to -1) would generate high r^2^ values. By default, the ASCAT data is provided as relative moisture and requires an adjustment with soil porosity values to provide correct absolute values. This adjustment was left out since it would not have influenced the result of the correlation analysis. Only significant (p < 0.05) correlations were taken into account. The dataset with the highest correlation was for each pixel selected and is referred to the per-pixel best dataset, from which spatial patterns could be identified and CCI could be compared to its individual products. A randomization test was performed to test if the number of pixels selected from each of the four datasets in the per-pixel best dataset was significantly different from a random. This was done with a spatial neighbourhood dependency built in. From the per-pixel best dataset, pairs of neighbouring pixels were selected randomly 100 000 times and the probability for each dataset having a neighbour with the same dataset identified as the best was recorded. We created 100 000 random per-pixel best datasets using the pre-calculated neighbour probabilities to assign the two neighbouring pixels, again selecting the pairs randomly. The distribution of the relative amount of pixels selected as the best was compared for each soil moisture dataset with the relative amount of pixels in the per-pixel best dataset to assess if the obtained result differed from random with the neighbouring dependency built in. The datasets were further evaluated by aridity index (AI) classes; arid (0.03 < AI < 0.2), semi-arid (0.2 < AI < 0.5), dry sub-humid (0.5 < AI < 0.65), and humid (AI > 0.65).

CCI was further analysed using a longer time series (2003–2014). This was done to provide an estimation of the dataset abilities to simulate NDVI for a longer temporal scale. The longer time series was used to re-calculate the correlation coefficient between modelled and measured NDVI. It was also expanded with the amplitude difference and Willmott’s Index of Agreement (IOA; [33]). The amplitude was calculated by taking the difference between maximum and minimum NDVI of the time period. IOA is a standardized model agreement measure where a value of 0.0 indicates no agreement and 1.0 indicates a perfect model agreement. Finally, the time series of CCI modelled NDVI and MODIS-NDVI for one pixel was shown. The selected pixel was the one that had the correlation closest to the average for all pixels. This was done to show the temporal agreement between the two series.

## Result

**Fig 1:**
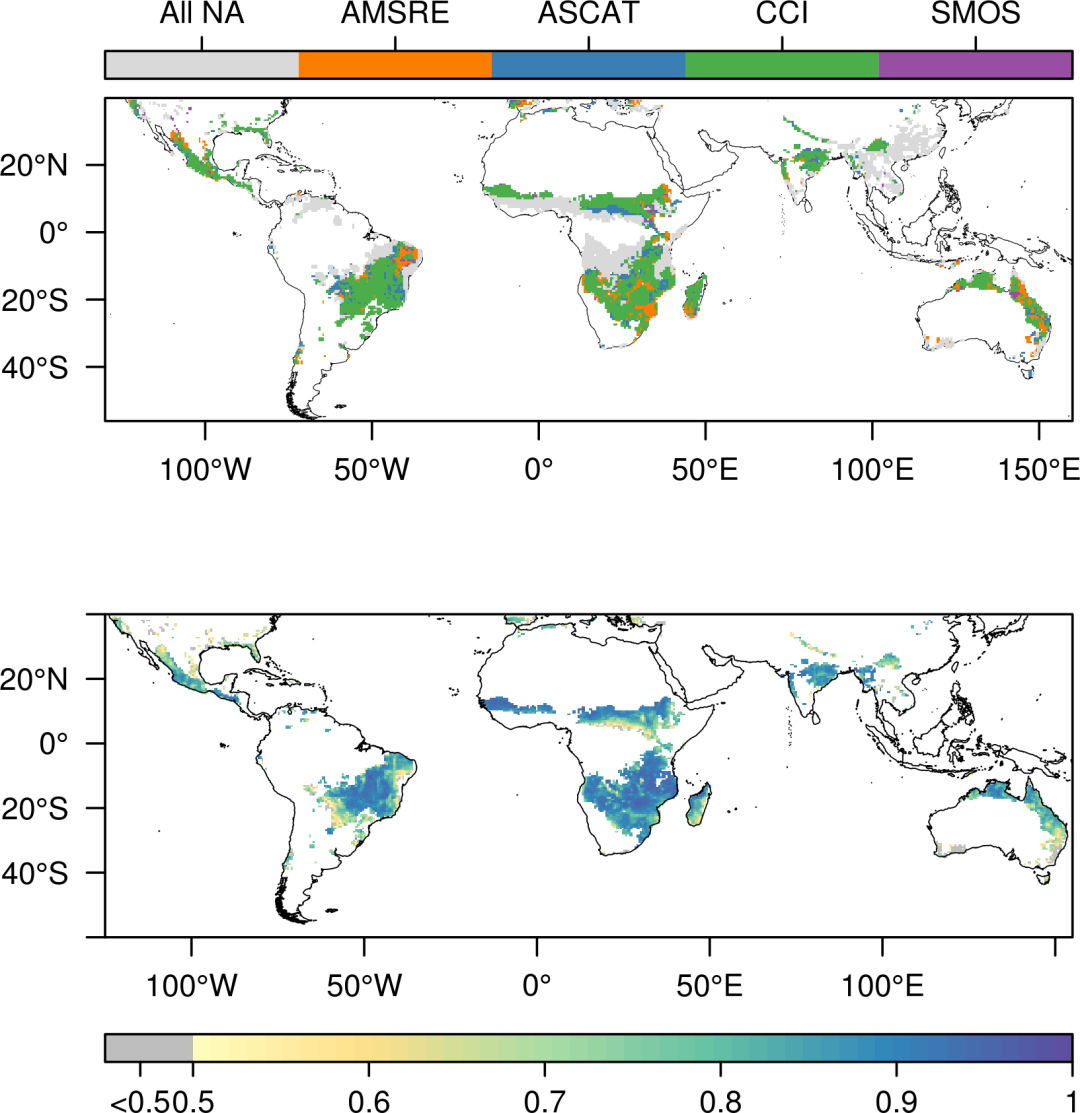
Best dataset analysis. Land borders have been created using data from thematicmapping.org. **Top:** Per-pixel best dataset identified as the one with the highest correlation between modelled NDVI and MODIS-NDVI using each of the four different soil moisture datasets. All NA indicate missing data in all datasets, no significant correlation to NDVI or correlation below 0.5 in all datasets. Data have for visual purposes been filtered with a 3x3 modal filter. **Bottom:** Best correlation (r) of modelled NDVI vs observed NDVI per-pixel. For each pixel, the highest correlation value shown as identified in the top panel. Non-significant or missing values are not shown in the plot. The grey color indicates a correlation below 0.5.

The average correlation coefficient (r) between MODIS-NDVI and NDVI modelled with each soil moisture dataset ranged from 0.63 to 0.81. We found a higher average correlation between MODIS-NDVI and CCI, r = 0.81, compared to the CCI individual products (AMSRE = 0.70, SMOS = 0.63, ASCAT = 0.74). Since the highest correlation varied by location, an option was to generate a dataset based on selecting the best method for each pixel. However, selecting the dataset with highest correlation per-pixel increased the average correlation by only 0.01 (r unit-less) compared to using CCI. In total, CCI was identified as the best dataset for 64% of the pixels, AMSRE for 15%, ASCAT for 16%, and SMOS for 5% (Fig 1, top panel). From the per-pixel best dataset (Fig 1, top panel) it was found that the probabilities of the same dataset being identified as best for two spatially adjacent pixels were 79% for CCI, 51% for AMSRE, 58% for ASCAT, and 32% for SMOS. Those proportions of pixels selected as the best for each dataset were significantly different (p<0.001) from selecting them randomly with the neighbor probabilities. This indicates that the found pattern is significantly different from random (p<0.001).

**Fig 2:**
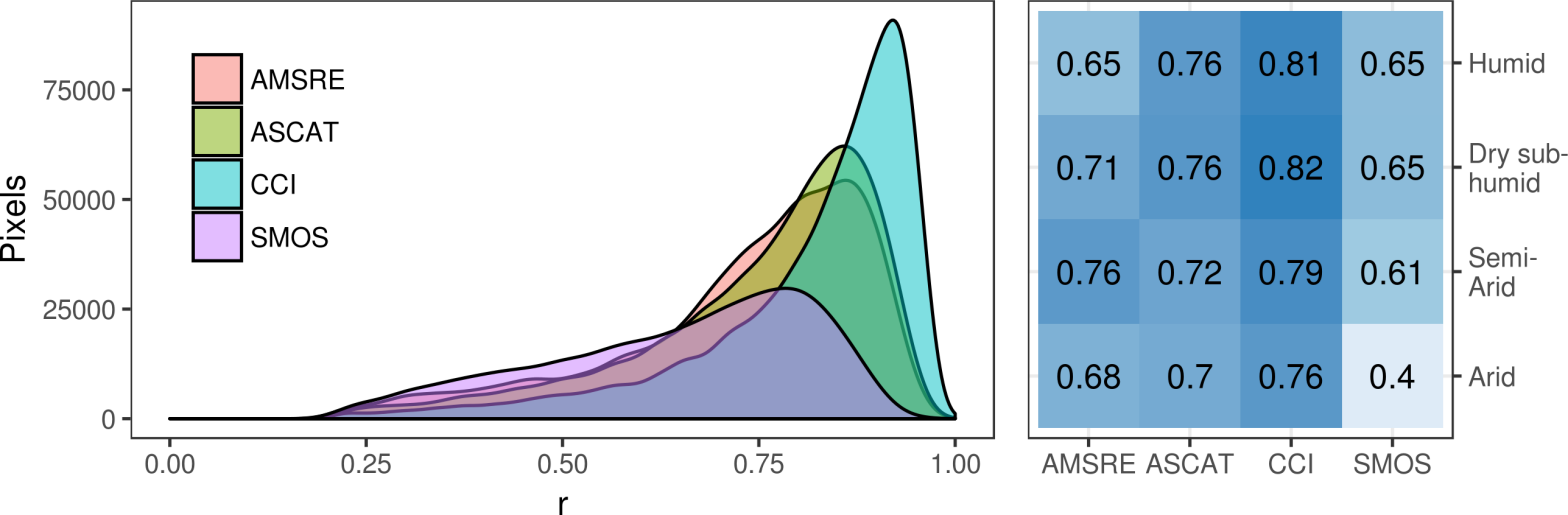
Correlation (r) between soil moisture modelled NDVI and MODIS-NDVI. **Left:** Distribution shown for all significant pixels. The few values below zero omitted for visual purposes. **Right:** Heat-map of the average correlation for each dataset used to model NDVI which has been divided into aridity classes; arid (0.03 < AI < 0.2), semi-arid (0.2 < AI < 0.5), dry sub-humid (0.5 < AI < 0.65), and humid (AI > 0.65). Average correlation values shown within heat-map grid.

We further found that NDVI modelled with CCI soil moisture had a higher average correlation than its individual products independent on aridity class (Fig 2 right). The shape of the correlation distribution revealed similarities between AMSRE and ASCAT and showed that CCI had the highest peak (Fig 2 left). SMOS differed by showing the least distinct peak. However, SMOS had the lowest number of pixels (n = 11241) with a significant correlation and non-missing data, to be compared to the other datasets which all included more than 16 000 pixels (AMSRE 16323, ASCAT 16091, and CCI 16644).

**Fig 3:**
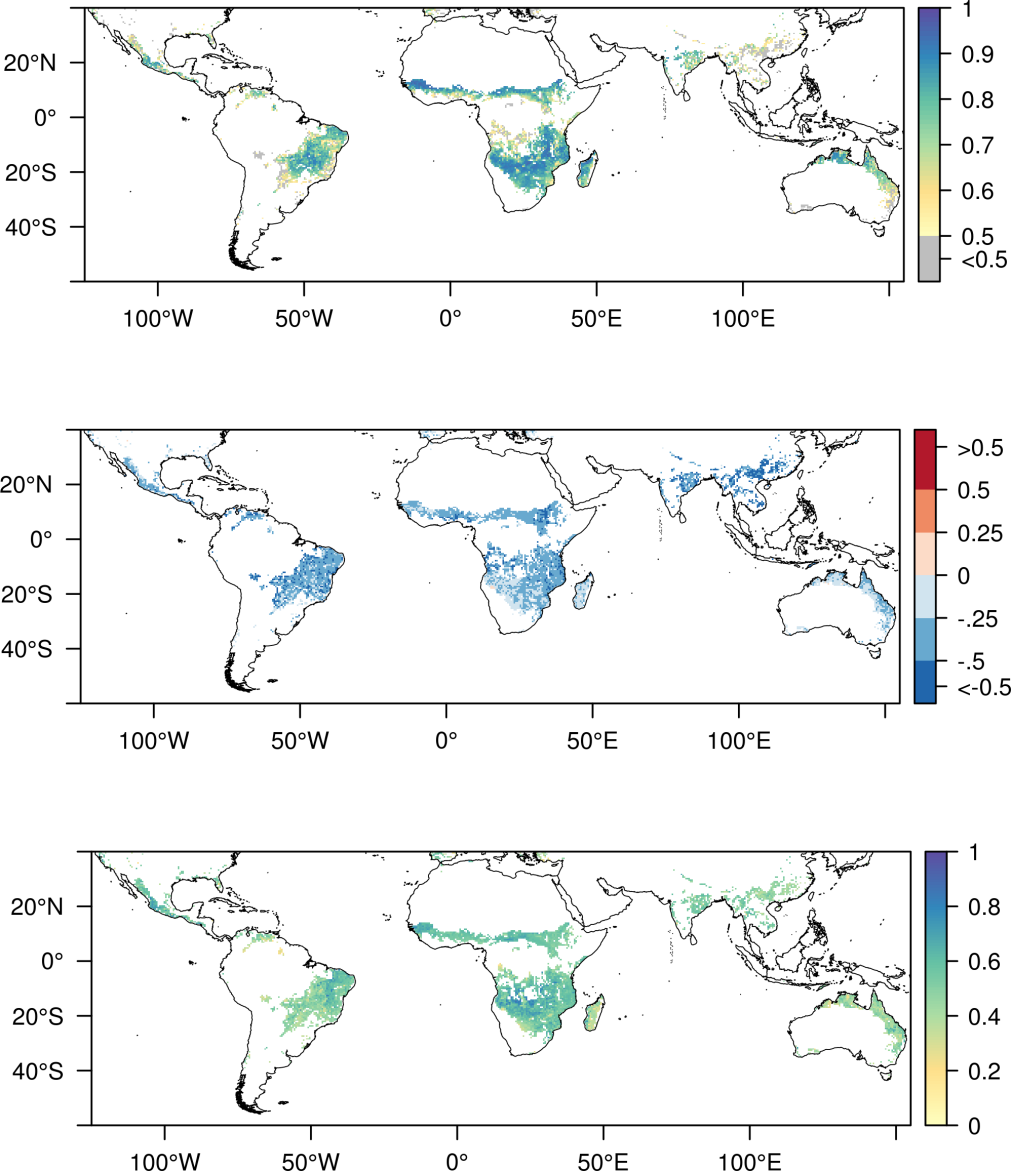
NDVI modelled with CCI compared to MODIS-NDVI for a longer temporal extent (2003 until 2014). Only pixels with significant correlation are shown. Land borders have been created using data from thematicmapping.org Top: correlation coefficient between modelled and measured NDVI. Middle: Amplitude difference between model and measured NDVI for the entire time period. (Negative indicates an underestimation of amplitude in the modelled NDVI). In total only 0.2% of the significant pixels had an amplitude difference above zero (red color). Bottom: Willmott Index of Agreement between modelled NDVI and MODIS-NDVI.

**Fig 4:**
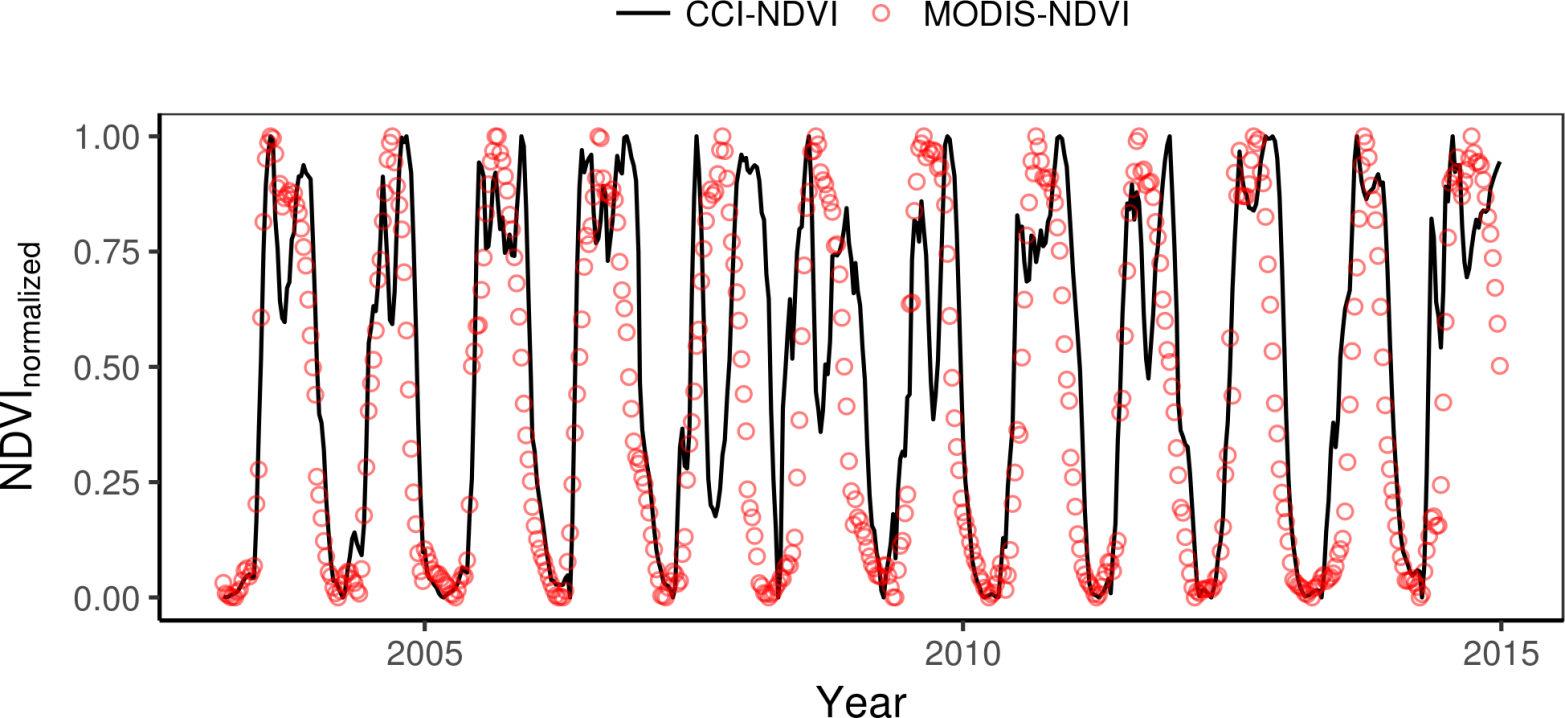
Normalized CCI modelled NDVI (CCI-NDVI) and MODIS-NDVI. Example time series shown for latitude 9.625, longitude 30.375. Pixel chosen since the correlation (r = 0.71) between MODIS-NDVI and CCI-NDVI) had the closest to the average correlation for all pixels. CCI-NDVI was for this figure filtered with the same Savitsky-Golay method as used for the MODIS-NDVI. Data was normalized between zero and one for each year separately.

Fig 3 shows the evaluation of the CCI soil moisture when applying the longer temporal extent (2003–2014). The correlation between CCI modelled NDVI and MODIS-NDVI was on average 0.71 and the amplitude difference showed an underestimation in the modelled NDVI (Fig 3) by on average -0.35. The IOA between CCI modelled NDVI and MODIS-NDVI for the longer time series was on average 0.52 and gave a similar spatial pattern as the correlation (Fig 3). The time series of CCI modelled NDVI and MODIS-NDVI for the example pixel (latitude 9.625, longitude 30.375, Fig 4) showed most inconsistencies for year 2007 were only the start of the growth period, increment in NDVI, was captured. It also gave a difference in the peak NDVI for seven years (2003, 2004, 2007, 2008, 2009, 2011, 2014) showing a strong decrease where MODIS-NDVI showed its peak (Fig 4). However, it showed for a majority of the years a good agreement in the seasonal cycles where start and end of season were well captured.

## Discussion

This study investigated the performance of the merged CCI soil moisture product from ESA to estimate NDVI for savannahs, globally. CCI was compared against three of its individual soil moisture datasets in order to evaluate their relative performance. We showed that the combined soil moisture product (CCI) gave the highest correlation to MODIS-NDVI compared to using one of the single products (ASCAT, AMSRE, or SMOS). The result is promising since CCI has a long temporal extent (1978 to 2014), which will allow for a greater use than one of the single products. Overall, CCI soil moisture only decreased the savannah average correlation by 0.01 compared to selecting the best out of the four datasets per-pixel.

The combined CCI product was for this study considered a separate dataset that could be compared against its individual products. This worked since the individual products were rescaled to a common scale when creating the dataset [20]. Similarly to our result, it has been shown that CCI has an equal or better performance compared to its included products, with exception of ASCAT [20]. However, by converting the soil moisture to NDVI we show that CCI outperforms all of its individual products for a majority of savannah grid cells. The difference to the study from Dorigo et al. [20] could be due to a very limited number of in-situ stations in the savannah region of that study, or on the conversion in our study to NDVI. Due to the simplicity of the model used to convert the remotely sensed soil moisture to NDVI we do not expect a perfect match to MODIS-NDVI. It does not account for a change in the relationship between soil moisture and NDVI due to the CO_2_ fertilization effect which has been shown to be stronger in arid regions [34]. However, we still regard the simulated NDVI a valid proxy since soil moisture has been proven to be the main driver of NDVI for savannah regions and since the model used is expected to give similar average result (r^2^ = 0.60±0.18), as found in this study, when applied to a larger savannah region [21].

It has previously been shown that the reliability of remotely sensed soil moisture decreases with increasing vegetation cover [35], and that the errors of SMOS increase with tree cover fraction [18]. We speculate that this is what is affecting the example pixel when it is showing a decrease in modelled NDVI when MODIS-NDVI is showing a peak for seven out of 12 years (Fig 4). However, we restrict our main analysis to correlations for savannahs we argue that this will not have any major effect on the validity of our result when comparing CCI to its individual products for estimating NDVI. Due to the chosen correlation technique we did not investigate the errors in absolute values but focused on accuracy of the temporal dynamics that the correlation shows. The temporal dynamics are important when estimating missing data since the datasets can be normalized to account for differences in absolute values. Furthermore, a time series of vegetation indices can relatively easily be converted into seasonality metrics such as start of growing season, peak season, end of growing season, and relative seasonal integrals of growth using existing software such as TIMESAT [36]. The level of detail in those phenology estimates is often dependent on spatial and temporal resolution of the used dataset. There exist studies dedicated to combining remotely sensed data from different sources to enhance spatial and temporal resolution [37, 38]. The result of this study is therefore important since it shows that there could be a potential to combine the NDVI modelled with CCI with other remotely sensed datasets to enhance the phenological information that can be acquired.

## Conclusion

We conclude that the combined CCI soil moisture dataset from the European Space Agency outperforms three of its individual products (ASCAT, AMSRE, and SMOS) when estimating savannah NDVI. When looking at the longer time period (2003–2014), CCI modelled NDVI for savannah shows an average correlation of 0.71 compared to MODIS-NDVI. This indicates an opportunity for remotely sensed soil moisture from CCI to be used with the phenological model to estimate savannah NDVI. These results demonstrate a potential for further development in using CCI soil moisture in combination with other remotely sensed datasets to enhance quality and estimation of savannah vegetation phenology.
